# Mental health of youth athletes during the second year of the COVID‐19 pandemic in Japan: A three‐wave cross‐sectional study

**DOI:** 10.1002/pcn5.70187

**Published:** 2025-08-16

**Authors:** Fumiaki Yano, Tomihisa Niitsu, Yusuke Nakata, Masaomi Iyo

**Affiliations:** ^1^ Department of Psychiatry Chiba University Graduate School of Medicine Chiba Chiba Japan; ^2^ Department of Child Psychiatry Chiba University Hospital Chiba Chiba Japan; ^3^ Department of Psychiatry International University of Health and Welfare Narita Hospital Narita Japan

**Keywords:** adolescent, COVID‐19, mental health, pandemic, youth athlete

## Abstract

**Aim:**

This study aimed to examine the mental health of Japanese youth athletes during the second year of the COVID‐19 pandemic and explore its associations with demographic and sport‐related factors.

**Methods:**

Three cross‐sectional online surveys were conducted at a public high school in Japan in Spring 2021 (baseline; *n* = 1022), Fall 2021 (*n* = 1104), and Spring 2022 (*n* = 1066). Mental health was assessed using the Kessler‐6 (K6), Patient Health Questionnaire‐9 (PHQ‐9), and Generalized Anxiety Disorder‐7 (GAD‐7) scales. Participants were categorized as youth athletes (national‐level competitors [NC] and local‐level competitors [LC]) or nonathlete students (NAS). Logistic regression was used to identify the mental health risk factors.

**Results:**

Compared to the baseline, youth athletes showed higher rates of poor mental health indicators in the second and third surveys. Among them, female athletes showed higher risks of depression and anxiety in both later surveys, whereas upper grade athletes had an increased risk of depression in the second survey. No consistent association was found between COVID‐19 infection history and mental health.

**Conclusion:**

Japanese youth athletes showed poorer mental health indicators at later points in the second year of the pandemic. Female and upper grade athletes may require targeted mental health support. Continuous monitoring is essential during extended public health emergencies.

## INTRODUCTION

The outbreak of the novel coronavirus disease (COVID‐19) rapidly evolved into a global pandemic, significantly affecting both physical and mental health worldwide. On March 11, 2020, the World Health Organization officially declared COVID‐19 a pandemic,^1^ prompting governments worldwide to implement large‐scale public health measures. In Japan, these measures included the closure of schools in March 2020, a government‐declared public health emergency in April 2020, and the issuance of stay‐at‐home and social distancing orders. Such measures caused social isolation, loss of school‐based support, and routine disruption, factors that are considered especially harmful to adolescents. Early in the pandemic, experts called for long‐term monitoring of its effects on youth mental health.^2^

In Japan, even during the second year of the pandemic (Spring 2021 to Spring 2022), although vaccination efforts expanded (by November 2021, over 77% had received at least one dose^3^) and restrictions eased worldwide, strict measures remained in place in schools, including silent lunches, mask use during physical education, and the cancellation of school events. This period in the study region also saw two declarations of a state of emergency (from January 8 to March 20, 2021, and from August 2 to September 29, 2021). These prolonged restrictions may have had a substantial impact on students' mental health.^4^ Among these students, youth athletes, who rely heavily on structured activities, physical activity, goal setting, and social interaction for their well‐being, may have been particularly vulnerable to negative mental health effects, potentially altering their athletic identity.^5^ Studies from the first year of the pandemic reported worsening mental health among youth athletes, with team sports participants, females, and older students identified as high‐risk groups.^6^, ^7^, ^8^ As the second year of the pandemic began and restrictions started to ease in many countries, research emerged examining how the resumption of sports affected youth athletes' mental health. In the United States, by May 2021, the return to athletic activities was associated with improvements in both mental health and physical activity.^9^ The continued restrictions in Japan, despite global easing at that time, raise questions about their prolonged impact on young athletes.

This study aimed to fill this knowledge gap by examining the mental health of Japanese youth athletes during the second year of the COVID‐19 pandemic. Based on these findings, we formulated the following hypotheses:

Hypothesis 1 (H1) Japanese youth athletes would report higher levels of mental health problems during the second year of the pandemic, compared to earlier periods.

Hypothesis 2 (H2) Female sex, participation in team sports, upper grade level, and a history of COVID‐19 infection are associated with greater mental health risks.

To test these hypotheses, we surveyed Japanese high school students, including youth athletes, and assessed their mental health using validated psychological measures.

## METHODS

### Participants

The impact of the COVID‐19 pandemic on mental health is influenced by various contextual factors, including regional differences, infection control policies, and the specific phase of the outbreak.^10^, ^11^ To minimize the influence of these confounding variables and focus on youth athletes' mental health under consistent conditions, we selected a single institution: High School A. This school is located in a metropolitan area and is an average‐sized public high school in Japan, known for its active sports clubs and nearly equal sex distribution. All enrolled students (approximately 1200 individuals aged 15–18 years, ranging from elite youth athletes to nonathlete students [NAS]) were invited to participate in each survey. Participation was voluntary, and no exclusion criteria were applied beyond absence or failure to provide consent. No further selection procedures were implemented, and participants did not receive any incentives or compensation.

### Study design and data collection

This study employed an anonymous, online, cross‐sectional design and was conducted at three time points: Spring 2021 (baseline survey, March 1–10, 2021), Fall 2021 (second survey, October 28 to November 2, 2021), and Spring 2022 (third survey, March 1–18, 2022). By each survey time, cumulative COVID‐19 cases in Japan were 0.35%, 1.37%, and 4.04%, respectively.^12^, ^13^, ^14^, ^15^

The survey was administered during homeroom periods across different days for each class. Students completed the approximately 10‐minute questionnaire using their personal smartphones via Questant (Macromill, Inc.). The survey URL was shared through Microsoft Teams, the school's main communication platform. Students who were absent on the day of their homeroom session also received the survey URL via Teams. Although the URL was not individualized, access was restricted after the designated response period to minimize duplicate submissions.

### Measures

#### Demographics and classification criteria

The survey collected demographic data, including sex, grade, and club affiliation. Club affiliation was categorized as sports clubs, cultural clubs, or no affiliation. Cultural clubs included activities related to the arts and academics, such as drama, music, science, and art.

For students in sports clubs, the competitive level was assessed using two indicators: past selection history and tournament participation. The selection history options, in descending order, were as follows: Japan national team, the Kanto regional (comprising Tokyo and surrounding prefectures in Eastern Japan) selection, prefectural selection, subprefectural block selection, and no selection. Tournament participation options, in descending order, were international, national, the Kanto regional, prefectural, and subprefectural block championships. Both indicators were treated as ordinal variables.

Based on these responses, the students were classified into three groups: national‐level competitors (NC), local‐level competitors (LC), and NAS (see Supporting Information for details). In the following sections, the term *youth athletes* refers collectively to NC and LC.

- NC included students who had participated in international or national tournaments or had been selected for the Japan national team, Kanto, or prefectural teams. In this context, the prefectural and Kanto selections were considered indicators of national‐level competitiveness.
- LC included students who had participated in subprefectural block or school‐level tournaments and whose selection history was below the prefectural level.
- NAS included students who were not classified as athletes, such as those who did not participate in sports activities or those involved in nonathlete support roles (e.g., equipment or administrative assistants in sports clubs).

#### COVID‐19‐related questions

To assess the impact of the COVID‐19 pandemic, participants were asked about their history of polymerase chain reaction (PCR) testing, as well as their own and their cohabiting family members' history of COVID‐19 infection.

#### Kessler‐6

Kessler‐6 (K6)^16^ is a six‐item scale designed to assess nonspecific psychological distress experienced over the past 4 weeks. Responses were recorded on a five‐point scale, ranging from “none” [0] to “very much” [4]. The total score ranged from 0 to 24. We used a cutoff score of 5 to identify cases of psychological distress.^16^ For this study, we employed the Japanese version of the K6, which has proven to be accurate and reliable.^17^ The Cronbach's *α* coefficient was 0.87 in the baseline survey, 0.87 in the second, and 0.90 in the third.

#### Patient Health Questionnaire‐9

Patient Health Questionnaire‐9 (PHQ‐9)^18^ is a nine‐item scale that assesses the severity of depressive symptoms experienced over 2 weeks on a four‐point scale, ranging from “none” [0] to “almost every day” [3]. The scores range from 0 to 27. A cutoff score of 10 was used to identify moderate‐to‐severe depressive symptoms.^18^ For this study, we employed the Japanese version of the PHQ‐9, which has shown accuracy and reliability.^19^ The Cronbach's *α* coefficient was 0.85 in the baseline survey, 0.86 in the second, and 0.87 in the third.

#### Generalized Anxiety Disorder‐7

Generalized Anxiety Disorder‐7 (GAD‐7)^20^ is a seven‐item scale that assesses the severity of anxiety symptoms experienced over 2 weeks on a four‐point scale, ranging from “none” [0] to “almost every day” [3]. The scores ranged from 0 to 21. A cutoff score of 10 was used to identify cases of moderate‐to‐severe anxiety.^20^ For this study, we employed the Japanese version of the GAD‐7, which has been proven to be accurate and reliable.^21^ The Cronbach's *α* coefficient was 0.86 in the baseline survey, 0.83 in the second, and 0.85 in the third.

### Statistical analysis

#### Group classification and descriptive statistics

The participants were classified into three groups: NC, LC, and NAS. Descriptive statistics, including frequencies (*n*) and percentages (%), were used to characterize the sample.

#### Assessment of sociodemographic differences

To examine sociodemographic differences across the three survey periods, we generated cross‐tabulation tables using the following demographic variables: sex, grade level, PCR test history, personal and cohabiting family members' history of COVID‐19 infection, and levels of athletic competition. Chi‐square tests of independence were performed for all the variables. The chi‐square test results were used to describe the sample characteristics across time points and did not inform the selection of covariates in the regression models.

#### Mental health assessment

K6, PHQ‐9, and GAD‐7 scores were calculated for each participant. The median and interquartile range (IQR) were determined for each group separately. The Shapiro–Wilk test indicated that the distributions of these scales were non‐normal; therefore, nonparametric statistical methods were used. Each score was dichotomized based on established cutoff values, with scores classified as either below (=0) or above (=1) the threshold.

#### Logistic regression analysis

To test Hypothesis 1, cross‐tabulations were created between group classification (NC, LC, and NAS) and dichotomized mental health scores (K6 ≥ 5, PHQ‐9 ≥ 10, and GAD‐7 ≥ 10). Chi‐square tests of independence were conducted to examine the differences between the groups. Binary logistic regression was performed separately for each survey wave, using NC as the reference group. Covariates included sex, grade level, PCR test history, and personal and cohabiting family members' history of the infection.

To test Hypothesis 2, logistic regression was conducted using data from youth athletes (NC and LC). Dichotomized K6, PHQ‐9, and GAD‐7 scores were the dependent variables. The independent variables included sex, grade level, PCR test history, personal and cohabiting family members' history of the infection, type of sport (team vs. individual), and location of activity (indoor vs. outdoor). The forced enter method was used for all models.

All analyses were conducted using IBM SPSS Statistics (version 29), with a two‐tailed significance level of *p* < 0.05.

## RESULTS

### Demographic data

The detailed demographic characteristics are presented in Table 1. In the baseline survey, of the 1201 students surveyed, 1133 responded (response rate: 94.3%). After excluding 111 incomplete or invalid responses, 1022 students were included in the analysis, corresponding to 85.1% of the total student population. A total of 228 participants (22.3%) were classified as NC, 395 (38.6%) as LC, and 399 (39.0%) as NAS. In the second survey, of the 1195 students surveyed, 1156 responded (response rate: 96.7%). After excluding 52 incomplete or invalid responses, 1104 students were included in the analysis, corresponding to 92.4% of the total student population. A total of 280 participants (25.4%) were classified as NC, 412 (37.3%) as LC, and 412 (37.3%) as NAS. In the third survey, of the 1198 students surveyed, 1090 responded (response rate: 90.1%). After excluding 24 incomplete or invalid responses, 1066 students were included in the analysis, corresponding to 89.0% of the total student population. A total of 242 participants (22.7%) were classified as NC, 415 (38.9%) as LC, and 409 (38.4%) as NAS.

**Table 1:** Respondents' characteristics in each survey time point.

Variable	Baseline, Spring 2021 (*n* = 1022)	Second, Fall 2021 (*n* = 1104)	Third, Spring 2022 (*n* = 1066)	*p*
Sex				0.316
Male	498 (48.7%)	593 (51.0%)	554 (52.0%)	
Female	524 (51.3%)	541 (49.0%)	512 (48.0%)	
High school grade				0.09
First	373 (36.5%	353 (32.0%)	366 (34.3%)	
Second	358 (35.0%)	379 (34.3%)	374 (35.1%)	
Third	291 (28.5%)	372 (33.7%)	326 (30.6%)	
History of PCR test				<0.001
Yes	380 (37.2%)	496 (44.9%)	609 (57.1%)	
No	642 (62.8%)	608 (55.1%)	457 (42.9%)	
History of personal COVID‐19 infection				<0.001
Yes	91 (8.9%)	145 (13.1%)	241 (22.6%)	
No	931 (91.1%)	959 (86.9%)	825 (77.4%)	
History of cohabiting family members' infection				<0.001
Yes	32 (3.1%)	102 (9.2%)	223 (20.9%)	
No	990 (96.9%)	1002 (90.8%)	843 (79.1%)	
Competition level				0.497
National‐level competitors (NC)	228 (22.3%)	280 (25.4%)	242 (22.7%)	
Local‐level competitors (LC)	395 (38.6%)	412 (37.3%)	415 (38.9%)	
Nonathlete students (NAS)	399 (39.0%)	412 (37.3%)	409 (38.4%)	

*Note*: Demographic data for survey participants are presented separately for all participants. Values are presented as numbers (frequency %). This table presents the *p*‐values derived from independence tests (chi‐square tests) conducted on cross‐tabulated data between the three points (Spring 2021, Fall 2021, and Spring 2022) and the participants' sociodemographic data (sex, grade, history of polymerase chain reaction test, and history of personal and cohabiting family member's COVID‐19 infection).

Abbreviation: PCR, polymerase chain reaction.

### Scores of psychological scales

**Figure 1:**
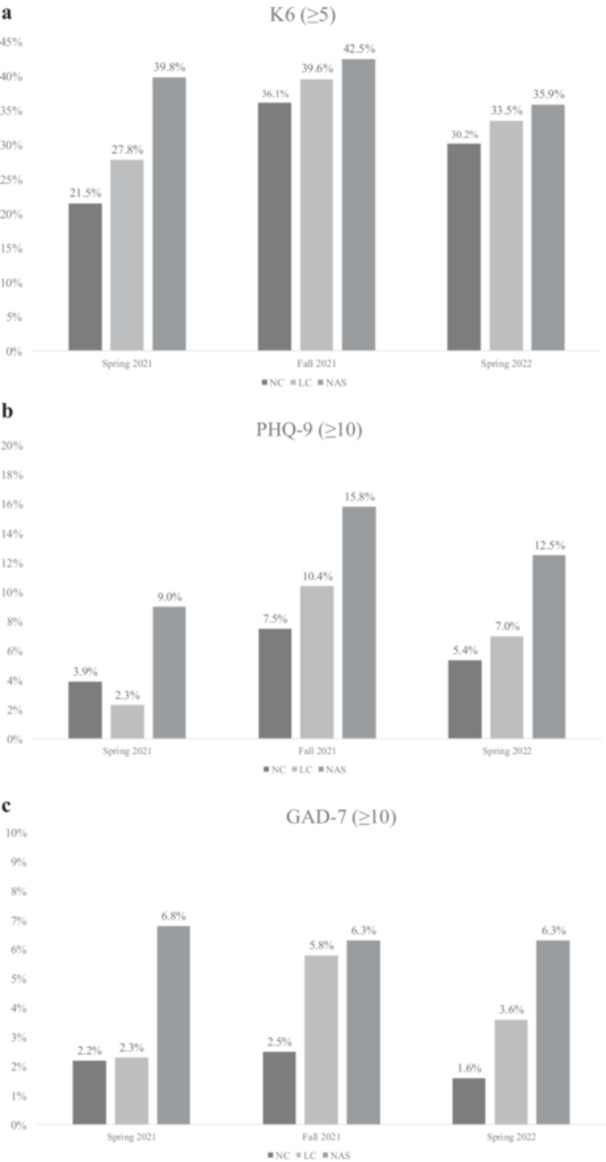
(a–c) Proportions of participants scoring above the cutoff on Kessler‐6 (K6), Patient Health Questionnaire‐9 (PHQ‐9), and Generalized Anxiety Disorder‐7 (GAD‐7) across three time points (Spring 2021, Fall 2021, and Spring 2022) for national‐level competitors (NC), local competitors (LC), and nonathlete students (NAS). A general trend was observed in which all groups showed higher proportions in Fall 2021 and Spring 2022 than in Spring 2021.

The proportion of participants scoring above the cutoff on the K6, PHQ‐9, and GAD‐7 is shown in Figure 1, and the detailed distribution of each scale score by median, IQR, and cutoff values is reported in Table 2.

**Table 2:** Psychological status of the groups.

	All participants	NC	LC	NAS	*p*_Adj_	Post hoc, OR (95% CI)
Baseline, Spring 2021 (*N*)	1022	228	395	399		
K6 score	2 (0–6)	2 (0–4)	2 (0–5)	3 (0–7)		
K6 (≥5)	318/1022 (31.1%)	49/228 (21.5%)	110/395 (27.8%)	159/399 (39.8%)	<0.001	NC versus NAS***, 2.2 (1.4–3.4)
PHQ‐9 score	2 (0–5)	2 (0–3)	2 (0–5)	3 (0–7)		
PHQ‐9 (≥10)	92/1022 (9.0%)	9/228 (3.9%)	9/395 (2.3%)	36/399 (9.0%)	<0.001	NC versus NAS***, 2.8 (1.3–6.3)
GAD‐7 score	1 (0–3)	0 (0–2)	0 (0–2)	1 (0–3)		
GAD‐7 (≥10)	41/1022 (4.0%)	5/228 (2.2%)	9/395 (2.3%)	27/399 (6.8%)	0.02	NC versus NAS***, 3.7 (1.2–10.9)
Second, Fall 2021 (*N*)	1104	280	412	412		
K6 score	4 (0–7)	3 (0–7)	3 (0–7)	4 (0–8)		
K6 (≥5)	439/1104 (39.8%)	101/280 (36.1%)	163/412 (39.6%)	175/412 (42.5%)	n.s.	
PHQ‐9 score	3 (1–6)	2 (1–5)	3 (1–5.75)	3 (0–7)		
PHQ‐9 (≥10)	129/1104 (11.7%)	21/280 (7.5%)	43/412 (10.4%)	65/412 (15.8%)	0.026	NC versus NAS*, 1.9 (1.1–3.2)
GAD‐7 score	1 (0–4)	1 (0–3)	1 (0–3)	1 (0–4)		
GAD‐7 (≥10)	57/1104 (5.16%)	7/280 (2.5%)	24/412 (5.83%)	26/412 (6.31%)	n.s.	
Third, Spring 2022 (*N*)	1066	242	415	409		
K6 score	2 (0–6)	2 (0–5)	3 (0–6)	3 (0–7)		
K6 (≥5)	359/1066 (33.7%)	73/242 (30.2%)	139/415 (33.5%)	147/409 (35.9%)	n.s.	
PHQ‐9 score	2 (0–5)	2 (0–4)	2 (0–5)	2 (0–6)		
PHQ‐9 (≥10)	93/1066 (8.72%)	13/242 (5.37%)	29/415 (7.0%)	51/409 (12.5%)	0.049	NC versus NAS*, 1.9 (1.0–3.7)
GAD‐7 score	1 (0–3)	0 (0–2)	1 (0–3)	1 (0–4)		
GAD‐7 (≥10)	45/1066 (4.22%)	4/242 (1.65%)	15/415 (3.61%)	26/409 (6.36%)	0.035	NC versus NAS*, 3.3 (1.1–9.8)

*Note*: The median (interquartile range) and number (frequency %) of participants above the Kessler‐6 (K6), Patient Health Questionnaire‐9 (PHQ‐9), and Generalized Anxiety Disorder‐7 (GAD‐7) cutoff scores are presented for all groups. Odds ratios (ORs), 95% confidence intervals (CIs), and adjusted *p*‐values (*p*_Adj_) were derived from logistic regression analyses adjusted for sex and polymerase chain reaction test history. The two covariates were identified based on chi‐square tests between the athletic competition level groups and sociodemographic variables, consistently observed across all three survey waves (Spring 2021, Fall 2021, and Spring 2022).

Abbreviations: LC, local‐level competitors; NAS, nonathlete students; NC, national‐level competitors; n.s., not significant.

*p < 0.05, **p < 0.01, and ***p < 0.001.

In the second and third surveys, youth athletes showed higher rates of poor mental health indicators compared to the baseline (Figure 1 and Table 2).

In the baseline survey, NC (21.5%) had a significantly lower rate of distressed students for the K6 scores compared to NAS (39.8%) with an odds ratio (OR) of 2.2 (95% confidence interval [CI]: 1.4–3.4, *p* < 0.001). Similarly, NC showed significantly lower rates of depression (3.9%) and anxiety (2.2%) based on PHQ‐9 and GAD‐7 scores, respectively, compared to NAS (9.0% and 6.8%, respectively), with ORs of 2.8 (95% CI: 1.3–6.3, *p* < 0.001) for depression and 3.7 (95% CI: 1.2–3.5, *p* = 0.02) for anxiety.

In the second survey, NC (7.5%) had a significantly lower rate of depressive students for the PHQ‐9 scores compared to NAS (15.8%) with an OR of 1.9 (95% CI: 1.1–3.2, *p* < 0.001), whereas no difference was found for the K6 and GAD‐7 scores among the groups.

In the third survey, NC showed significantly lower rates of depression (5.4%) and anxiety (1.7%) based on PHQ‐9 and GAD‐7 scores, respectively, compared to NAS (12.5% and 6.4%, respectively), with ORs of 1.9 (95% CI: 1.0–3.7, *p* = 0.049) for depression and 3.3 (95% CI: 1.1–9.8, *p* = 0.035) for anxiety, whereas no difference was found for the K6 score among the groups.

### Risk factors of mental health problems

Table 3 shows the associations between demographic variables and the binary indicators defined by whether scores on the K6, PHQ‐9, and GAD‐7 exceeded their respective cutoff values in youth athletes. In the baseline survey, third‐year athletes were associated with less distress on the K6 score compared to first‐year athletes (OR = 0.51, *p* = 0.011). A history of PCR testing showed a higher association with depression on the PHQ‐9 score (OR = 3.92, *p* = 0.005). In the second survey, female athletes were associated with a higher risk of depression and anxiety (OR = 2.43, *p* = 0.002; OR = 4.83, *p* < 0.001, respectively). Additionally, second‐ and third‐year athletes were associated with a higher risk of depression compared to first‐year athletes (OR = 2.33, *p* = 0.027; OR = 2.99, *p* = 0.005, respectively). In the third survey, female athletes were significantly associated with higher risk of distress, depression, and anxiety (OR = 1.42, *p* = 0.048; OR = 2.82, *p* = 0.002; OR = 3.16, *p* = 0.019, respectively). As observed in the baseline, third‐year athletes were associated with less distress compared to first‐year athletes (OR = 0.61, *p* = 0.026).

**Table 3:** Risk factors for youth athletes' mental health problems.

	Distress (K6 score ≥ 5)			Depression (PHQ‐9 score ≥ 10)			Anxiety (GAD‐7 score ≥ 10)		
	OR	95% CI	*p*	OR	95% CI	*p*	OR	95% CI	*p*
*Baseline, Spring 2021*									
Sex			n.s.			n.s.			n.s.
High school grade						n.s.			n.s.
First	ref								
Second	1.26	0.83–1.91	0.29						
Third	0.51	0.31–0.86	0.011						
History of PCR testing			n.s.						n.s.
Yes				ref					
No				3.92	1.50–10.27	0.005			
History of personal COVID‐19 infection			n.s.			n.s.			n.s.
History of cohabiting family members’ infection			n.s.			n.s.			n.s.
Type of sports			n.s.			n.s.			n.s.
Location of activity			n.s.			n.s.			n.s.
*Second, Fall 2021*									
Sex			n.s.						
Male				ref			ref		
Female				2.43	1.40–4.20	0.002	4.83	2.12–10.98	<0.001
High school grade			n.s.						n.s.
First				ref					
Second				2.33	1.10–4.92	0.027			
Third				2.99	1.39–6.46	0.005			
*Third, Spring 2022*									
Sex									
Male	ref			ref			ref		
Female	1.42	1.00–2.00	0.048	2.82	1.47–5.43	0.002	3.16	1.21–8.25	0.019
High school grade						n.s.			n.s.
First	ref								
Second	0.90	0.60–1.34	0.59						
Third	0.61	0.39–0.94	0.026						

*Note*: Analyses were conducted only among student athletes (only national‐level competitors and local competitors). Odds ratios (ORs), 95% confidence intervals (CIs), and *p*‐values are based on binary logistic regression analyses using the forced enter method. The dependent variables were binary indicators, defined by whether scores on the Kessler‐6 (K6), Patient Health Questionnaire‐9 (PHQ‐9), and Generalized Anxiety Disorder‐7 (GAD‐7) exceeded their respective cutoff values.

Abbreviation: PCR, polymerase chain reaction.

## DISCUSSION

### Summary of results

This study investigated the mental health status of Japanese youth athletes during the second year of the COVID‐19 pandemic. We conducted surveys at three points: the beginning of the second year (baseline, Spring 2021), the midpoint (second, Fall 2021), and the end (third, Spring 2022). To control for contextual variability in pandemic‐related stress, we focused on a single public high school. The main findings are as follows:

i. Youth athletes showed higher rates of poor mental health in the second and third surveys than at baseline.
ii. At baseline, NC reported lower scores on distress, depression, and anxiety compared to NAS. NC also showed significantly lower depression scores than NAS in both the second and third surveys, and lower anxiety scores in the second survey.
iii. Female youth athletes were more likely to report depression and anxiety in the second survey, and showed higher rates of distress, depression, and anxiety in the third.
iv. No significant risk of mental health problem was found in any type and location of sport activity.
v. Third‐year athletes showed a lower risk of distress in both spring surveys (baseline and third), but in the fall survey (second), second‐ and third‐year athletes had a higher risk of depression compared to first‐year athletes.
vi. At baseline, youth athletes without a history of COVID‐19 testing were associated with a higher risk of depression, whereas no other association among COVID‐19‐related factors and mental health problems was observed in all the surveys.

### Summary of hypotheses and findings

*Japanese youth athletes would report higher levels of mental health problems during the second year of the pandemic compared to earlier periods.*

This hypothesis was supported. Youth athletes showed higher proportions of poor mental health indicators in the second and third surveys than at baseline. These results suggest that a greater number of youth athletes reported mental health difficulties as the pandemic persisted.

*Female sex, participation in team sports, upper grade level, and a history of COVID‐19 infection are associated with greater mental health risks.*

This hypothesis was partially supported. Female sex and upper grade level were associated with higher risks of mental health problems. However, no significant differences were found based on the type of sport or COVID‐19 infection history.

### Discussion of key findings

Among youth athletes, the second and third surveys revealed higher rates of poor mental health indicators than at baseline. These results suggest that youth athletes may have experienced poorer mental health during the second year of the pandemic. This highlights the need for continued psychological monitoring, even after the partial resumption of sports activities. In Japan, strict restrictions on adolescent activities, such as holding events without spectators and cancelling major competitions (e.g., the National High School Championships and National Sports Festival in 2021), persisted into the second year of the pandemic, which may have contributed to these findings. A recent report analyzing sports‐related COVID‐19 clusters indicated that most outbreaks were linked to non‐exercise settings, such as indoor gatherings and shared meals, rather than physical activity itself. This suggests that blanket suspension of sports, driven by concerns over infection, might have inadvertently exacerbated mental health challenges among youth athletes, as such measures may not be the most effective prevention strategy for COVID‐19 transmission.^22^

At baseline, NC reported significantly lower scores on distress, depression, and anxiety compared to NAS, and they also showed lower levels of depression and anxiety in the second survey, and lower levels of depression in the third survey. These findings suggest that despite the overall decline in mental health during the second year of the pandemic, NC consistently had a better psychological status than NAS. Previous findings indicated that NC showed higher resilience in early 2021, including both internal traits and external support, such as family, school, and peers.^23^ Although most resilience factors had relatively modest effects during the pandemic, family‐level resilience emerged as particularly effective in buffering pandemic‐related stress.^24^ These findings suggest that family support may play a key role in sustaining mental health among NC. Furthermore, while sports activities had only partially resumed for NC, it is likely that their physical activity levels remained higher than those of NAS. Physical activity has confirmed beneficial effects on adolescent mental health,^25^ and studies conducted during the pandemic reported that youth who remained physically active tended to show better mental health outcomes.^26^

In the second survey, female youth athletes were associated with risks of depression and anxiety, whereas these risks of female were extended to all mental health problems in the third survey. These findings reinforce the pattern that female youth athletes experienced poorer mental health in the later surveys. Female youth athletes are more prone to eating disorders and disordered eating, which are strongly associated with depression and anxiety.^27^ Although training gradually resumed in the second year, pandemic‐related disruptions may have contributed to increased concerns regarding food control, body composition, and weight. A study of adult female athletes reported that disordered eating behaviors, including preoccupation with food, dietary restriction, and fear of weight gain, worsened during the pandemic,^28^ and similar vulnerabilities may exist among younger athletes. However, the specific mechanisms underlying poor mental health in female youth athletes remain poorly understood and require further investigation.^8^

No significant differences in mental health were observed between participants in individual and team sports. However, previous studies have indicated that team sport athletes are at a greater risk for mental health problems during the pandemic, partly because individual athletes were more likely to continue training while adhering to social distancing guidelines.^6^, ^7^, ^8^ As this study did not assess training volume, it remains unclear whether differences in training opportunities contributed to the observed results.

In the baseline and third surveys conducted in spring, the third‐year students (final year students in Japanese high schools) were associated with lower levels of psychological distress compared to first‐year students. In contrast, in the second survey conducted in the fall, both second‐ and third‐year students were associated with a higher risk of depression. This suggests that older students experienced reduced stress in spring as graduation approached (the academic year runs from April to March in Japan) but showed greater depressive symptoms during the academic year. Many youth athletes faced uncertainty regarding whether competitions would be held or whether they would be forced to withdraw because of COVID‐19. For upper grade students, this unpredictability may have been particularly stressful, as each event could represent their final opportunity to compete. This finding aligns with previous research from the United States, where older high school athletes reported greater mental health deterioration during the pandemic.^7^

At baseline, youth athletes without a history of COVID‐19 testing were associated with a higher risk of depression, suggesting that untested students may have experienced greater uncertainty and psychological distress in response to pandemic‐related events. Notably, in the months leading up to the baseline survey, the school had experienced a temporary closure due to a cluster outbreak, which may have contributed to heightened psychological strain, particularly among those who had not undergone testing and may have felt more uncertain about their health status. No associations were found between COVID‐19‐related variables and psychological assessment scales in the second and third surveys. This may be due, in part, to the relatively low infection rates in our sample (8.9% in the baseline, 13.1% in the second, and 22.6% in the third). In contrast, previous studies involving collegiate and professional athletes reported higher infection rates (29%–44.3%) and found that a history of COVID‐19 infection was associated with a greater rate of depression symptoms.^29^, ^30^

### Limitations

This study had some limitations. First, it was an online, self‐administered survey; therefore, mental health assessments may not reflect formal clinical diagnoses. Second, although three separate cross‐sectional surveys were conducted, the study did not adopt a longitudinal design, which limited the ability to examine trends or shifts at the individual level across time points. Third, we did not collect information on participants' current or past mental or physical health conditions. The presence of such conditions may have influenced their mental health status during the survey periods. Fourth, we did not collect detailed information on students' training durations or frequencies. This limited our ability to interpret the relationship between physical activity levels and mental health, particularly given the various restrictions imposed on sports clubs during the pandemic. Lastly, we did not assess participants' socioeconomic background, which may have significantly influenced mental health outcomes not only among youth athletes^7^ but also among adolescents in general.^26^

## CONCLUSION

This study examined the mental health status of Japanese youth athletes during the second year of the COVID‐19 pandemic. A greater proportion of youth athletes reported poor mental health in the context of prolonged restrictions that were still in place at the time. These findings highlight the need for ongoing psychological monitoring and support, particularly for high‐risk groups such as female and upper grade youth athletes. Future research should focus on developing and evaluating effective interventions to support the mental health of youth athletes during and after public health emergencies.

## AUTHOR CONTRIBUTIONS

Fumiaki Yano contributed to all aspects of the study, including the study design, data collection, statistical analysis, and manuscript drafting. Tomihisa Niitsu was involved in the study design and statistical analysis and provided critical revisions to the manuscript. Yusuke Nakata contributed to the study design and grant acquisition. Masaomi Iyo contributed to the study design and coordinated the research. All authors approved the final version of the manuscript.

## CONFLICT OF INTEREST STATEMENT

The authors declare no conflicts of interest.

## ETHICS APPROVAL STATEMENT

This study was approved by the Ethics Review Committee of the Graduate School of Medicine, Chiba University (Approval No. 4113).

## PATIENT CONSENT STATEMENT

An explanatory document regarding this study was distributed to the students' teachers and parents. We obtained written consent to conduct the study from the school principal. The screen for starting the questionnaire explained the purpose and method of the study, the results if they participated or did not participate, ethical considerations, and handling of the results. A consent question was also presented. No incentives were offered to students to encourage participation in the study.

## CLINICAL TRIAL REGISTRATION

N/A.

## Supporting information

Supporting Information.

## Data Availability

The data that support the findings of this study are available on request from the corresponding author. The data are not publicly available due to privacy or ethical restrictions.
