# Supplementary material for: Mental health of youth athletes during the second year of the COVID‐19 pandemic in Japan: A three‐wave cross‐sectional study
Source: PCN Rep. 2025 Aug 16;4(3):e70187. doi: 10.1002/pcn5.70187 (PMC12357596; doi:10.1002/pcn5.70187)
Supplement: Supplementary file 1 — Supporting Information. [file PCN5-4-e70187-s001.docx]

**Supplementary 1. Factors that provided the basis for grouping**

| Variable | Baseline, Spring 2021  (n=1022) | Second, Fall 2021  (n=1104) | Third, Spring 2022  (n=1066) |
| --- | --- | --- | --- |
| Type of club activities |  |  |  |
| Sports | 692 (67.7%) | 744 (67.4%) | 717 (67.3%) |
| Culture | 223 (21.8%) | 240 (21.7%) | 224 (21.0%) |
| None | 107 (10.5%) | 120 (10.9%) | 125 (11.7%) |
| Type of sports |  |  |  |
| Team | 343 (33.6%) | 398 (36.1%) | 368 (34.5%) |
| Individual | 280 (27.4%) | 294 (26.6%) | 289 (27.1%) |
| Others | 399 (39.0%) | 412 (37.3%) | 409 (38.4%) |
| Location of sports |  |  |  |
| Outdoor | 369 (36.1%) | 280 (25.4%) | 267 (25.0%) |
| Indoor | 254 (24.9%) | 412 (37.3%) | 390 (36.6%) |
| Others | 399 (39.0%) | 412 (37.3%) | 409 (38.4%) |
| Degree of contact |  |  |  |
| Contact sports | 213 (20.8%) | 247 (22.4%) | 237 (22.2%) |
| Non-contact sports | 410 (40.1%) | 445 (40.3%) | 420 (39.4%) |
| Others | 399 (39.0%) | 412 (37.3%) | 409 (38.4%) |
| Best results in sports |  |  |  |
| International | 6 (0.6%) | 9 (0.8%) | 5 (0.5%) |
| National | 156 (15.3%) | 162 (14.7%) | 145 (13.6%) |
| Kanto region | 84 (8.2%) | 159 (14.4%) | 135 (12.7%) |
| Prefecture | 283 (27.7%) | 267 (24.2%) | 264 (24.8%) |
| City | 94 (9.2%) | 95 (8.6%) | 108 (10.1%) |
| Others | 399 (39.0%) | 412 (37.3%) | 409 (38.4%) |
| Selection history |  |  |  |
| National team | 3 (0.3%) | 8 (0.7%) | 7 (0.7%) |
| Kanto region team | 12 (1.2%) | 31 (2.8%) | 29 (2.7%) |
| Prefecture team | 136 (13.3%) | 148 (13.4%) | 136 (12.8%) |
| City team | 79 (7.7%) | 123 (11.1%) | 94 (8.8%) |
| None | 393 (38.5%) | 382 (34.6%) | 391 (36.7%) |
| Others | 399 (39.0%) | 412 (37.3%) | 409 (38.4%) |
